# Nitrous Oxid with Air or with Oxygen

**Published:** 1900-04

**Authors:** 


ARTICLE III.

### NITROUS OXID WITH AIR OR WITH OXYGEN.

At a meeting of the New York State Medical Association held in New York, October 26th, 1899, Dr. Thomas L. Bennett, of New York, presented a paper on the administration of the above-named combinations. He stated that the inhalation of nitrous oxid was often followed by headache, dizziness, persistent nausea, or a tendency to yawn for twenty-four hours or more. He had been unable to find on record any disturbance of the heart, lungs, and kidneys, and from his own experience was sure that such results must be extremely rare. The prolonged administration of this agent was difficult, because the stages were exceedingly short and sharply marked. On the other hand, if considerable air was admitted, the patient would not pass quietly into deep narcosis, but would present marked signs of excitement. Hence it was necessary to admit air in small quantities. Deep narcosis was present after about one minute, and if all had been rigidly excluded, there would be also marked asphyxia. Mixtures of nitrous oxid with pure oxygen afforded an ideal combination; and they were best made in practice, by the use of Hewitt's apparatus. All changes in the proportion of gases must be made gradually, as sudden changes were apt to interfere with the smoothness of the narcosis. This method undoubtedly afforded the safest and best form of nitrous oxid anæsthesia, possessing all of its advantages and none of its disadvantages. It was practically impossible to move the patient after the anæsthesia had been started, without disturbing the narcosis In a long administration of the mixed gases it was not uncommon to consume five dollars' worth of the gases. The abdomen was apt to remain rigid even when the narcosis was deep, and hence this method was not ordinarily well suited to abdominal operations. The greatest indications for this anæsthesia were to be found: (1) in those in whom ether or chloroform could not be used without special danger; (2) in operations so short as to render the effects of ether or chloroform out of proportion to the results; (3) in patients who had previously suffered extremely from ether or chloroform, and had in consequence a great deal of them. This anæsthetic agent had been found especially serviceable in the following cases: tooth extractions, incisions of abscesses and sinuses or division of strictures, curettings of various kinds, stretching in orthopedic cases, breaking up adhesions in joints, removal of tonsils, doing major dressings, in the aged and in those presenting lesions of the heart or kidneys.

Dr. Golden said that he was positive that the degree of cyanosis depended almost entirely on the experience and skill of the administrator. The longest period that he had maintained narcosis with gas had been two hours, and with the mixture of gas and oxygen one hour and a half.

Dr. De Lancey Rochester said that in most general hospitals it would be difficult to find a sufficiently experienced administrator on the house staff, and in rural practice it was hard to secure the gases.—*Medical Record.*

---

ARTICLE IV.

